# Attenuated behavioral interpersonal synchrony in autistic adults is not explained by perception of timing

**DOI:** 10.1038/s41598-025-05395-1

**Published:** 2025-06-20

**Authors:** Afton M. Bierlich, Nanja T. Scheel, Jana C. Koehler, Carola Bloch, Irene Sophia Plank, Christine M. Falter-Wagner

**Affiliations:** 1 Department of Psychiatry and Psychotherapy, LMU University Hospital, LMU Munich, Munich, Germany; 2 Department of Psychiatry and Psychotherapy, Faculty of Medicine and University Hospital Cologne, University of Cologne, Cologne, Germany

**Keywords:** Autism, Perceived interpersonal synchrony, Event timing perception, Behavioral interpersonal synchrony, Naturalistic social interactions, Human behaviour, Psychology

## Abstract

Attenuated interpersonal synchrony (IPS) has been shown between autistic individuals and their interaction partners; however, the mechanisms of this attenuation remain unclear. One possibility could lie in perceiving the timing of others’ behaviors. The present study aimed to relate the behavioral production of IPS with the perception of temporal dynamics of social interactions and event timing perception in autistic and non-autistic adults. Autistic and non-autistic participants engaged in naturalistic conversations with a non-autistic stranger, who was naïve to the participant’s diagnostic status. Behavioral IPS was computed using automatic video-based analysis. Participants reported their experiences of perceived IPS with the partner, as a measure of the perceived temporal dynamics of the social interaction. A perceptual simultaneity task measured the perception of event timing in a nonsocial context. Bayesian linear mixed models were used to evaluate the effects of perceived IPS ratings and simultaneity thresholds on behavioral IPS. Expectedly, behavioral IPS was reduced for dyads including an autistic adult. Neither perceived IPS ratings, nor simultaneity thresholds, were associated with reduced behavioral IPS for dyads with or without an autistic adult. These findings hint that attenuated behavioral IPS may not result from atypical perceived timing of others’ behaviors or event timing perception.

**Supplementary Information:**

The online version contains supplementary material available at 10.1038/s41598-025-05395-1.

## Introduction

When we interact with other people, we tend to synchronize our behaviors with those of our interaction partner to establish smooth interactions^1^. This phenomenon, known as interpersonal synchrony (IPS), mediates social exchange, promotes prosocial behaviors^2–4^, facilitates rapport^5–7^, influences our impressions of people and interactions^8,9^, and strengthens social bonds^10–12^. Autism Spectrum Disorder (ASD) is a neurodevelopment condition that is characterized by social reciprocity and communication difficulties, as well as restricted and repetitive behaviors^13^. Atypical social reciprocity can be operationalized in terms of reduced IPS, which has been shown in autism^14^. Considerable literature has often investigated behavioral IPS, and the attenuation thereof in autism, specifically in the motor domain^15,16^. Accordingly, multiple studies have shown reduced motor IPS in naturalistic contexts, such as in typical conversations^17–20^ and clinical interviews^21–23^. Dyads including an autistic individual have shown reduced behavioral IPS compared to non-autistic dyads, regardless of the interaction partners’ diagnostic status^17^.

Importantly, the underlying mechanisms of attenuated behavioral IPS in autism are not understood. Differences in perceptual processes, namely perceiving the timing of other’s behaviors, could account for behavioral IPS attenuation. Altered timing processes have been suggested as a contributing factor underlying social interaction difficulties in autism^24–29^. Altered processing of stimulus timing has been shown in autistic individuals with varying performance across different temporal domains, such as relative timing, interval timing, temporal event-structure coding, temporal generalization^30–35^, and across sensory domains^26,27,35–37^. Disrupted timing processes could affect how autistic individuals perceive the timing of others’ behaviors. However, mixed findings (e.g.,^34,38,39^, and for a review, see^26^) across studies and paradigms demonstrate both differentiated (enhanced or attenuated) and comparable timing capabilities, suggesting a nuanced pattern of time processing in autism.

Although many timing domains work in concert, the perception of event timing is particularly relevant when investigating IPS to ascertain how the basic detection of temporally aligned stimuli might translate to more complex temporal processes for synchronizing nonverbal behaviors. Some studies^33,40^ have found enhanced visuo-temporal resolution for autistic compared to non-autistic individuals, meaning that autistic individuals had lower thresholds for detecting simultaneity (but also see^34^). Moreover, superior visual resolution in autistic individuals has been linked with atypical non-verbal communication^33^. As such, and in line with the social timing hypothesis of autism^29^, it is plausible that differentiated perception of event timing could lend to differences in perceiving the timing of another person’s behaviors, and subsequently reduced behavioral IPS. Thus, it is pertinent to investigate how the temporal dynamics of social interactions is perceived to determine the perceptual contribution to the observed behavioral attenuation thereof in autism.

Notably, the perceptual basis of the temporal dynamics of social interactions, specifically in terms of IPS, has only recently been explored. Few studies have investigated how IPS is subjectively perceived when one is part of and observing social interactions. Some findings have demonstrated that typical observers reported perceiving lower IPS when interacting with less synchronous virtual partners^41^. Similarly, increased perceived togetherness has also been reported when individuals listened to a virtual interaction with manipulated simultaneity and regularity^42^. Increased ratings of perceived IPS have also been shown when observing social interactions with higher behavioral IPS^43^. Moreover, evidence further hints that perceiving rudimentary synchronous behaviors may be intact in autism. Autistic individuals have reported similar experiences of perceived IPS to non-autistic individuals when interacting with virtual partners^44,45^. Notably though, these paradigms are limited as individuals either interacted with a virtual partner whose responses were perceptually rudimentary and temporally predictable or observed others’ interactions without being involved in these interactions. Natural social interactions, like in face-to-face conversations, are much more complex in terms of the social information that needs to be processed. Accordingly, autistic and non-autistic individuals may perceive the temporal dynamics of social interactions differently when they engage in such naturalistic interactions. Thus, in order to ascertain whether perceptual mechanisms underlie attenuated behavioral IPS, we must establish how the temporal dynamics are perceived in naturalistic social interactions.

To discern whether the differences in perceiving the timing of others’ behaviors underlies attenuated behavioral IPS in autism, the present study aimed to link behaviorally produced IPS with the perception of temporal dynamics of social interactions and event timing processes in autism through a relevant context, namely collaborative naturalistic social interactions with another person. We expected behavioral IPS would be reduced for dyads including an autistic individual in such interactions. Further, differences in behavioral IPS would be associated with perceived temporal dynamics of social interactions and explored this relationship with perceived event timing processes. An association of timing perception measures with differentiated behavioral IPS between dyads with and without autistic participants would indicate that altered perceptual timing mechanisms are, at least in part, responsible for behaviorally attenuated IPS observed in autism.

## Methods

This study was part of a larger experimental testing session including fMRI studies reported elsewhere^44,46^. The tasks were pre-registered (https://osf.io/cw7n4), and additional behavioral analyses not reported here may be reported elsewhere. A power analysis was conducted for the larger project. Specifically, a power analysis (effect size = 0.31^41^, power = 0.99, alpha level = 0.05 based on a repeated measures ANOVA) yielded a minimum sample size of 25 participants per diagnostic group for analysis. Part of this data was used in a student monography^47^. The Ethics Committee of the Medical Faculty at the LMU University Hospital Munich approved this study (No. 20-1050) and was conducted in compliance with the Declaration of Helsinki^48^. Participants provided informed consent and were monetarily compensated for their participation. Custom Python (v3.8) scripts were used to present the tasks and collect the data, and preprocessing and analysis was carried out in RStudio (v4.1.3)^49^.

### Participants

We recruited 62 individuals (33 autistic, 29 non-autistic) via the LMU University Hospital Munich, as well as through local and regional channels. Individuals with a confirmed diagnosis of Autism Spectrum Disorder (F84.5, F84.0, F84.1), in accordance with the ICD-10^50^, were included in the autistic sample. Individuals without reported psychiatric diagnoses were included in the non-autistic sample. Participants were between 18 and 60 years old, had an IQ of 70 or above, normal or corrected-to-normal vision, and no reported neurological conditions. The Culture Fair Intelligence Test (CFT-20-R^51^) and Mehrfachwahl-Wortschatz-Intelligenztest (MWT-B; a multiple-choice vocabulary test^52^) assessed nonverbal and verbal IQ, respectively. Autism-like traits and motor coordination difficulties were evaluated via German translations of the Autism Spectrum Quotient (AQ^53^) and Adult Dyspraxia Checklist (ADC^54^). Rapport was evaluated using a questionnaire merged from Cacioppo et al.^41^ and Koehler et al.^18^. Participants took part in two additional MRI tasks on the same day, which are not reported here. Data from one participant (autistic) was excluded due to an IQ below 70.

The final sample consisted of 32 autistic (10 identified as female, 22 identified as male) and 29 non-autistic (11 identified as female, 18 identified as male) individuals. A sample characterization is reported in Table 1.

**Table 1:** Means and standard deviations are reported for each group, as well as Bayes factor (*BF*_*10*_) from a bayesian Mann–Whitney U test (^a^) or a bayesian independent samples t-test (^b^).

	Autistic	Non-autistic	BF_10_
Age ^a^	35.16 ± 10.52	34.31 ± 11.68	2.595
CFT-20-R ^b^	110.19 ± 21.27	119.21 ± 17.78	0.984
MWT-B ^a^	110.47 ± 12.29	117.03 ± 14.37	0.025
AQ ^a^	35.06 ± 6.95	15.52 ± 6.43	1.287 × 10^15^
Rapport ^a^	5.31 ± 1.18	5.57 ± 0.76	0.399

### Experimental design and setup

#### Conversational task

A conversational paradigm was used to mimic a naturalistic setting, in which participants conversed with an unacquainted interaction partner. The interaction partner was a confederate (university students or members of the research group) for logistical reasons, who was naïve to the participant’s diagnosis. Confederates were not explicitly trained for engagement in the interactions and received the same instructions as participants upon engaging in the task. Each confederate (*n* = 10) interacted with a nearly balanced number of autistic and non-autistic individuals (Supplementary Information 1). Participants and confederates were not matched for gender; they were assigned based on their respective availability for the testing session. A Bayesian contingency table demonstrated no credible differences between groups for the dyad compositions of the same gender and of different genders (*BF*_*10*_ = 0.311; non-autistic dyads: 17 different-gender, 12 same-gender; mixed dyads: 18 different-gender, 14 same-gender). A composite rapport rating (Cronbach’s α = 0.86) evaluated participants’ affiliative experiences with the interaction partner which were comparable for both groups (Table 1).

Each dyad was asked to talk about two conversational topics: (i) plan a five-course menu including foods that both interactants disliked (meal-planning task) and (ii) discuss their hobbies and interests (hobbies task). The meal-planning topic is a highly dynamic and collaborative task that promotes cooperative exchange and turn-taking, which has been commonly used for this purpose throughout the literature^17,18,55,56^. In contrast, the hobbies task served as a baseline task that does not require coordinated reciprocity. One person could speak about their hobbies for the entire conversation, which could result in longer periods of monologue speech and decreased turn-taking. A previous study using only a topic suggestion, and therefore not enforcing coordinated reciprocity, found no differences in behavioral IPS between dyads with and without autistic individuals^57^. Each conversation lasted 10 min.

All testing sessions took place in the same room with a stable background and artificial lighting conditions. The participant and confederate sat opposite of each other at a table, approximately 190 cm apart. A plexiglass pane separated the interactants to accommodate COVID-19 regulations. One camera (C922 Pro HD Stream Webcam, Logitech) was positioned 2.4 m from the table to capture a side view of the interaction. Participants and confederates wore headset microphones (t.bone earmic 500, Thomann GmbH) that were connected to the same audio recorder (Zoom H4n, Zoom Sound Service GmbH). Video and audio were synchronously recorded on a laptop using PsychoPy^58^. Audio data were subsequently omitted due to sporadic technical challenges during data acquisition (e.g., inseparable audio tracks, recording failures, cut-out audio).

#### Perceptual simultaneity task

A perceptual simultaneity judgement task was used to assess individuals’ event timing perception via their simultaneity thresholds in a nonsocial context. Participants judged whether two bars appeared simultaneously or not in a two-alternative forced-choice design. The task consisted of five blocks with 52 trials each, plus one practice block of ten trials that were randomly selected. Onsets of the bars were manipulated in steps of 8.33 ms, beginning from a difference of 0 ms to 100 ms (simultaneous onset asynchrony; SOA). The appearance of the bars onsets was manipulated by gradient color changes in five equal increments (10%) from black to grey using the rgb255 color space as implemented with the PsychoPy package^58^. The first bar onset appeared on the right or left side of the fixation cross with equal probability. All trials were randomly presented twice within each block. Stimuli were presented on a 120 Hz Acer monitor. Participants were seated in a quiet testing chamber, and the monitor was positioned approximately 40 cm away.

### Procedure

After completing several questionnaires, either the conversational tasks or the perceptual simultaneity task (counterbalanced) commenced. For the conversational task, an experimenter brought in the interaction partner (confederate). The participant and interaction partner were equipped with head set microphones, and the camera view was manually adjusted. They were first instructed to discuss either the hobbies or meal-planning topics (counterbalanced). The experimenters started the recordings and left the room during the conversation. This procedure was repeated for the second conversational topic. Following the second conversation, the interaction partner was escorted to another room. The participant then answered six questions about their rapport with the partner (Supplementary Information 2), and one question about the perceived synchrony that they experienced in the interaction (‘Wie synchron war die Kommunikation zwischen Ihnen und Ihrem Partner?’; ‘How synchronized was the communication between you and your partner?’). Ratings were evaluated using a 7-point Likert scale (1: not at all; 7: very much). They additionally were asked whether the video recording and plexiglass influenced their behavior (Supplementary Information 3). A mean composite rapport rating, using the six rapport questions, was later computed to evaluate participants’ affiliative experiences with the interaction partner. The question regarding participants’ experience of perceived synchrony constituted the perceived IPS rating. For the perceptual simultaneity task, a subset of participants (*n* = 34) completed the task on a separate day due to technical issues at the original session, resulting in a smaller sample for the computer task.

### Data preprocessing

#### Behavioral IPS

Dyadic behavioral movement IPS was measured via video recordings of the interactions. Frame-by-frame pixel changes were extracted using Motion Energy Analysis (MEA)^59^. The filtering threshold was set to eight as determined by visual inspection. Head and body region of interest (ROI) were defined in MEA for the participant and the confederate. The head ROI included the head and neck, and the body ROI included the shoulders, arms, hands, torso, and thighs. Concatenating the head and body ROI produced a total ROI for each individual. The head ROI was taken to represent behavioral IPS, given the reported differences in head movement in clinical populations during social interactions^17,22,56^ and the reliable computation above chance level compared with pseudo-synchrony.

Further preprocessing was conducted using the rMEA package^60^. MEA outputs were scaled, and IPS was computed for each ROI using lagged window cross correlations (30-second windows, a 5-second lag, 15-second overlapping increments, Z-transformed, absolute values). A peak picking method was adopted to maintain the variability in behavioral IPS across the interaction^61,62^ The maximum cross-correlation value (i.e., the peak) was taken within each window, and the average of the peaks across all windows were taken as a measure of behavioral IPS.

Pseudo-synchrony values were computed and comparatively evaluated to ensure that the behavioral IPS values were above chance level for each ROI. Dyadic behavioral IPS in the head region was decisively above chance level and used for the analyses. Dyadic motion quantity was computed from the absolute values of the overall movement within a dyad and was included as a covariate in the model. The computation, analysis, and visualizations of pseudo-synchrony and motion quantity are reported in Supplementary Information 4.

#### Perceptual simultaneity thresholds

Response accuracies were calculated as the number of simultaneous responses in each SOA condition. For each participant, the guess rate was calculated as the percentage of non-simultaneous responses for the simultaneous condition (i.e., SOA-0). Data from participants with a guess rate of 50% or higher were excluded from further analysis (*n* = 3). Bias correction was performed for each participant: P*adj*(x) = P(x)/P(0), where P(x) is the percentage of simultaneous responses for asimultaneous conditions, and P(0) is the percentage of simultaneous responses for the simultaneous condition. Following bias correction, a logistic curve, implemented with the quickpsy package^63^ and default settings, was fit across all SOAs for each participant to calculate the threshold (i.e., simultaneity threshold) and steepness of the curve (i.e., response criterion). Data from one participant was additionally excluded as an outlier considering a threshold exceeding two standard deviations of the group mean. The resulting sub-sample included 30 participants (17 autistic, 13 non-autistic).

### Data analysis

Bayesian linear mixed models, with sum-coding contrasts and a shifted log-normal likelihood distribution, were conducted using the brms package^64^ to evaluate the influence of the perceived temporal dynamics of social interactions (as indexed by perceived IPS ratings) and event timing perception (as indexed by simultaneity thresholds) on dyadic behavioral IPS from a collaborative naturalistic social interaction (as indexed by the meal-planning task). Two models were developed as only a sub-sample was available for the simultaneity thresholds. Considering that altered motor functions are sometimes reported in autism^65^ and have been associated with deficits in joint action synchronization^66^, measures of dyadic motion quantity, self-reported dyspraxia symptoms (as indexed by the ADC), as well as baseline behavioral IPS from a naturalistic social interaction not requiring coordinated reciprocity (as indexed by the hobbies task), were included as covariates in the models.

For model development, prior predictive checks, checks for computational faithfulness, and an assessment of model sensitivity were implemented using simulation-based calibration with the SBC package (version 0.2.0.9000)^67^ (Supplementary Information 5). We generated 500 simulated data sets to create prior predictive plots. The model was then run on the simulated data sets with four Markov chains, 40,000 iterations (20% warm-up), and initial values set to 0.1. Rhat scores (< 1.01), divergent transitions (ca. 20%), and posterior ranks were visually inspected before running the model on the true data. Posterior predictive checks (Supplementary Information 6) were visualized using the bayesplot package (version 1.10.0)^68^ before evaluating the significance of the estimated parameters using the brms *hypothesis* function. Parameter estimates with a posterior probability greater than 95% for the group predictor and a posterior probability greater than 97.5% for all other predictors were considered credible effects.

The first model (*N* = 61) included three categorical predictors: group (mixed dyads, non-autistic dyads), perceived IPS ratings, and their interaction. Dyadic motion quantity, individual ADC scores as an index of self-reported dyspraxia symptoms, and behavioral IPS from the hobbies task (behavioral IPSh) were entered as continuous covariates. Perceived IPS ratings and dyadic motion quantity were scaled and centered. ADC scores and behavioral IPS from the hobbies task were log-transformed before being scaled and centered. For completeness purposes, we ran the reverse model (with behavioral IPS from the hobbies task as the outcome) and reported it in Supplementary Information 7. A random intercept was included for confederates. We aimed to construct a maximal model including a random intercept for conversation task order and group as a random slope for each. Ultimately, we had to simplify the model due to divergence issues (> 20% of the simulated datasets had divergent transitions) but kept the intercept for confederate as a random effect. We deemed the inclusion of confederate important for explaining additional variance, as confederates interacted with both autistic and non-autistic participants. Weakly informative priors were chosen as they demonstrated wide but plausible potential values in the data-agnostic prior predictive distributions. The intercept priors were set with a normal distribution considering a mean of − 1 (i.e., log(0.368)) with a standard deviation of 0.25, and the sigma prior with a mean of 0.2 and a standard deviation of 0.1. The beta priors were centered at 0 with a standard deviation of 0.5, and the random effects priors were centered at 0 with a standard deviation of 0.2. The non-decision time parameter was set at 0.05 with a standard deviation of 0.15.

A second model was conducted (*N* = 30) which additionally included simultaneity thresholds. The same procedure, priors, and model parameters were used as with the first model. There were issues with the model fit (ca. 35% divergent transitions) indicating that the model was too complex considering the small sample, which should be considered when interpreting the results.

We diverge from the preregistered models as we aimed to use a more parsimonious approach which combines the assessment of behavioral IPS and perceived IPS ratings. The preregistered models are included in the Supplementary Information 8.

An exploratory analysis further assessed whether a particular model best explained the data. Using leave-one-out cross-validation, models with all possible combinations of the predictors (*N* = 32 models), except the interaction term of perceived IPS rating by group that was not included, were compared using the loo package (version 2.7.0)^69^. An expected log predictive density was computed for each model using the *loo* function. Model comparison was evaluated by the difference between the sum of the expected log predictive density using the *loo_compare* function.

Lastly, group comparisons were conducted for the sample characterization (Table 1) and model predictors. Bayesian independent samples t-tests were conducted (BayesFactor::ttestBF)^70^ when assumptions were met, and Bayesian Mann–Whitney U-tests (DFBA::dfba_mann_whitney)^71^ were used when normality was violated. Bayes factors were interpreted according to Jeffrey’s scheme^72^, and we consider all Bayes Factors that are at least moderate (*BF*_*10*_ > 3) to be credible evidence in favor of the alternative hypothesis.

## Results

**Fig. 1:**
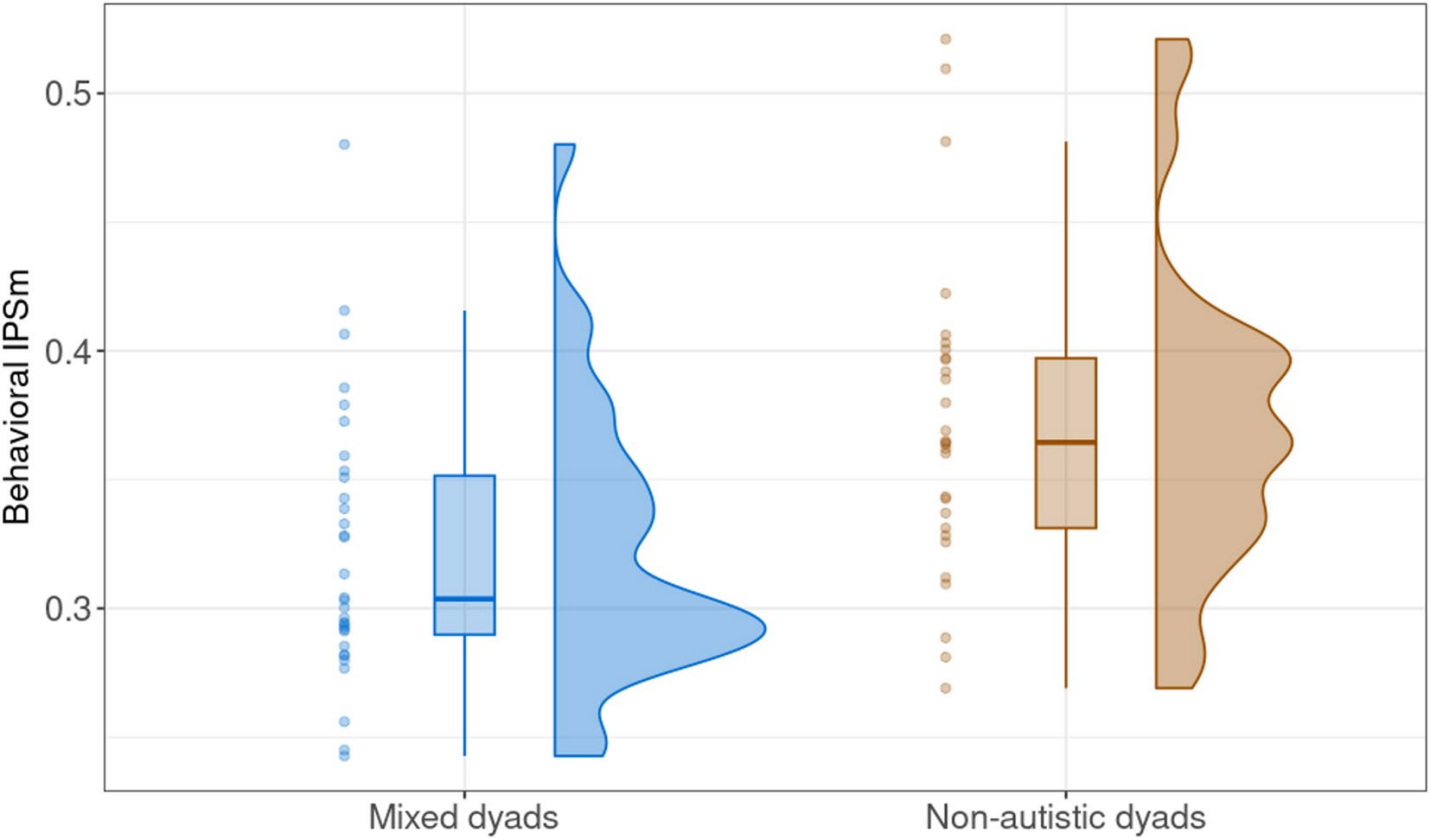
Raw behavioral IPS for mixed dyads (*n* = 32) and non-autistic dyads (*n* = 29) in the meal-planning task.

**Fig. 2:**
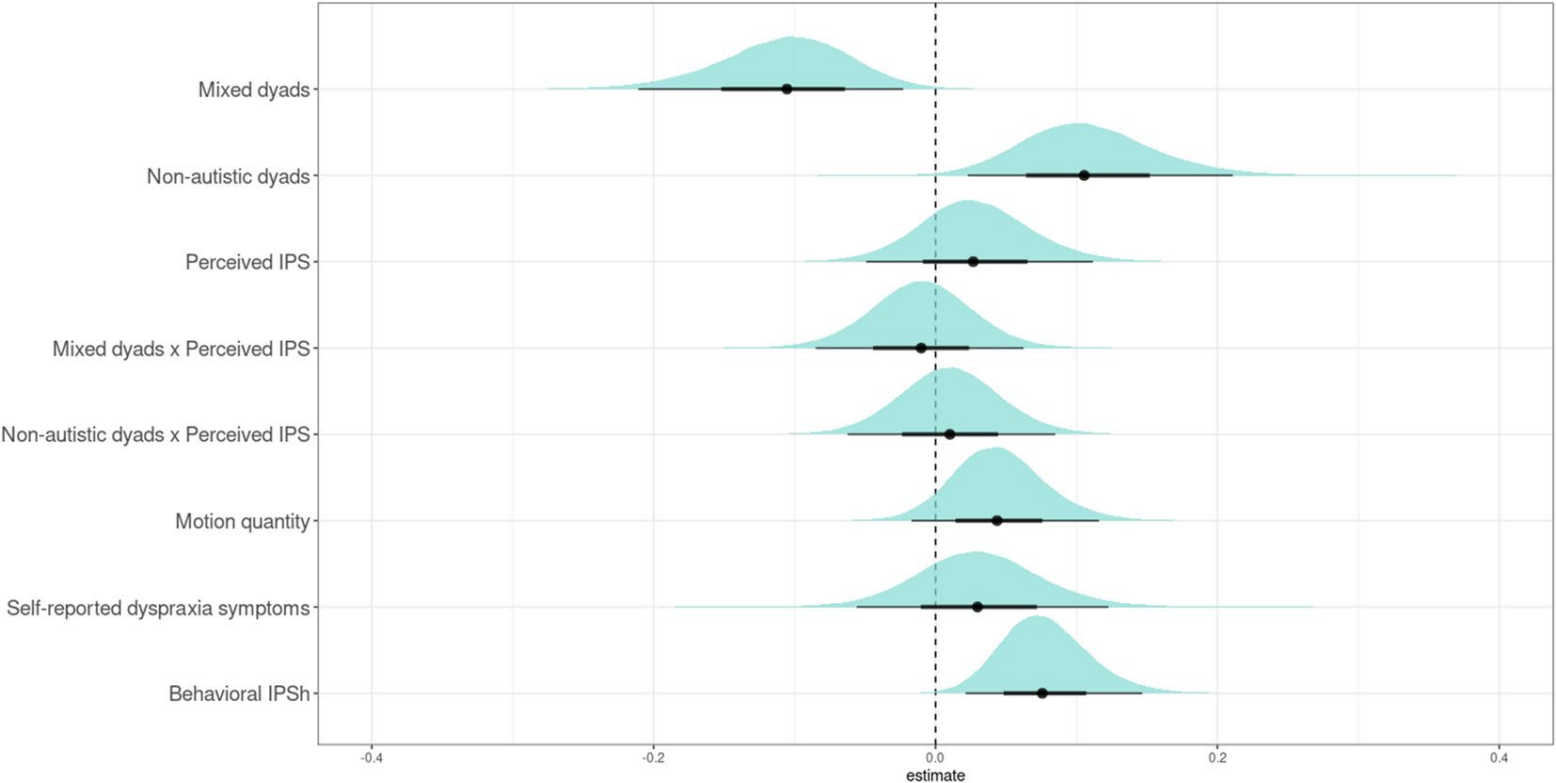
Posterior predictive distributions of the estimates for each predictor: group, perceived IPS ratings, their interaction, motion quantity, self-reported dyspraxia symptoms (ADC), and behavioral IPS from the hobbies tasks (behavioral IPSh). The lines reflect the 95% (thin) and 66% (thick) intervals of the distributions. The dotted line reflects the zero grand average estimate. Credible effects are visualized as those that do not cross the zero grand average estimate. Mixed dyads elicited less behavioral IPS than non-autistic dyads. Behavioral IPS from the baseline hobbies task had a positive association with behavioral IPS in the collaborative meal-planning task. Perceived IPS ratings, its interaction with group, motion quantity, and self-reported dyspraxia symptoms had no clear effect on behavioral IPS.

In the model that excluded simultaneity thresholds, there was an effect of group on behavioral IPS (Fig. 1; *estimate* = − 0.11, *posterior probability* = 0.99), as expected. Dyads including an autistic participant produced significantly less behavioral IPS than non-autistic dyads (mixed dyads M ± SD: 0.32 ± 0.05; non-autistic dyads: 0.37 ± 0.06). Behavioral IPS from the hobbies task was highly associated with the meal-planning task (*estimate* = 0.08, *posterior probability* = 1.00). Notably, no other predictors revealed a credible effect on behavioral IPS (Fig. 2). Contrary to our expectations, this indicates that behavioral IPS was not highly associated with the perceived IPS ratings, nor the included covariates of motion quantity or self-reported dyspraxia symptoms. Table 2 reports the group comparisons of the model predictors, and visualizations are depicted in Supplementary Information 9.

Model comparisons yielded that a model including group and behavioral IPS from the hobbies task best explained the data, which supports the effects observed from the model. This suggests that the mechanisms captured at present are not highly explanatory of behavioral IPS in a dynamic, collaborative task and hint that alternative mechanisms may rather underlie attenuated IPS in autism. A full table of the model comparisons is reported in Supplementary Information 10.

In the model that included simultaneity thresholds using the smaller sample, no predictors showed a credible association with behavioral IPS, suggesting that behavioral IPS was not highly associated with event timing. Visualizations of the estimates are depicted in Supplementary Information 11.

**Table 2:** Means and standard deviations of the raw scores are reported for each group for all predictors, except perceived IPS ratings, which includes the median and standard error. Bayes factors (*BF*_*10*_) are reported for each group comparison. ^a^ Bayesian Mann-Whitney U test. ^b^ Bayesian independent samples t-test.

	Autistic	Non-autistic	BF_10_
Perceived IPS ratings ^a^	5 ± 0.33	6 ± 0.20	7.100 × 10^− 4^
Simultaneity thresholds ^a^	51.42 ± 17.06	48.71 ± 15.83	2.527
Motion quantity ^b^	47.87 ± 10.62	49.65 ± 9.99	0.316
Self-reported dyspraxia symptoms ^a^	56.09 ± 18.10	17.93 ± 8.21	2.252 × 10 ^15^
Behavioral IPSh ^a^	0.29 ± 0.04	0.29 ± 0.04	0.460

## Discussion

In light of the growing literature emphasizing altered interpersonal synchrony (IPS) of nonverbal behaviors in autism^14–16^, this study sought to link the perception of temporal dynamics of social interactions, in terms of subjectively perceived IPS, and event timing perception, in terms of simultaneity thresholds, to behaviorally attenuated IPS in naturalistic social interactions including autistic and non-autistic individuals. We replicated previous findings^17,19,22^ showing reduced behavioral IPS for dyads including an autistic individual during a collaborative exchange in a naturalistic conversation. Yet, perceptual characteristics were not predictive of behaviorally attenuated IPS.

One possibility of why perceived IPS was not associated with behavioral motor IPS is that participants might have considered different behavioral modalities to inform their perception of the temporal dynamics of the interaction, given that IPS can exist at multiple behavioral levels^14^. For instance, recent studies report on IPS in naturalistic interactions including autistic individuals, not only in the motor domain, but also with facial expressions^18,73^, speech^55,74^, as well as physiological^75^ and neural^76–78^ patterns. Thus, it is possible that other behavioral features informed how participants perceived the temporal dynamics of social interactions subsequent to the social interactions. Moreover, the findings suggest that event timing perception may not be associated with behavioral IPS and does not explain the attenuation thereof in autism. However, this warrants cautious interpretation, in which the link between event timing perception and behavioral IPS needs to be bolstered within a larger sample. Nonetheless, this initial finding, together with the null link between perceived IPS and behavioral IPS, hint that timing perception, in general and of others’ behaviors specifically, likely may not account for attenuated behavioral IPS in autism.

Furthermore, it is plausible that we have not yet captured the primary mechanism(s) that contribute to attenuated behavioral IPS consistently observed in autism. Other cognitive mechanisms, such as social attention, multisensory integration as well as mentalization and mutual adaptation, have been suggested as components mediating behavioral IPS, and differentiated processing of such cognitive mechanisms in autism could lend to attenuated behavioral IPS^79^. We speculate how such cognitive mechanisms might explain the attenuation of behavioral IPS presently shown.

Atypical social attention is one potential mechanism that could have contributed to attenuated behavioral IPS in the present study. Studies have shown that autistic individuals attend less or in a delayed manner to the face/head region when viewing social stimuli^80–83^, specifically attending less to the eye region of an interaction partner^84–87^. Reduced attention toward the head region in the present study would suggest that autistic participants may not have detected the timing of the interaction partner’s movements simply because their gaze was not directed at this region. In line with this idea, the head region has been shown to be of particular relevance for motor IPS in autism^17,22^. Relatedly, increased variability in gaze patterns when perceiving social stimuli^88^ and in decoding strategies of nonverbal communication^85^ has also been shown for autistic individuals. As we did not capture gaze patterns during the interaction, we cannot infer whether reduced attention to the head region, nor variability in gaze pattern and decoding strategies, contributed to the attenuation of behavioral IPS observed. Future studies should include eye-tracking to capture gaze and fixation patterns to determine whether different aspects of atypical social attention may account for attenuated behavioral IPS in interactions including autistic individuals.

Atypical multisensory integration is another candidate mechanism that could lend to attenuated behavioral IPS in autism. Natural social interactions, such as a face-to-face conversation, include verbal and nonverbal behaviors. Altered multisensory integration in autism has been widely shown (for a meta-analytic review, see^89^). Fewer studies have investigated multisensory integration in autism using speech stimuli. Some studies^19,36,90–93^ have shown reduced temporal acuity of speech and visual stimuli (but also see^39,94^). Noel et al.^19^ further linked multisensory processing to behavioral IPS in naturalistic interactions of adolescents with and without autism. The authors found that autistic individuals had lower temporal acuity of multisensory inputs, which was not correlated with behavioral IPS in autistic individuals as it was for non-autistic individuals^19^. Noel et al.^19^ postulated that autistic individuals may not integrate the timing of the multisensory information into their higher order representations. If so, reduced temporal acuity of verbal and non-verbal behaviors could influence how multisensory information is integrated into higher-order cognitive processes, such as action planning and execution, which could subsequently lend to reduced behavioral IPS. Accordingly, future studies could extend this line of investigation by assessing how multisensory integration of audio-visual information, such as that observed in typical conversations, is linked with behaviorally attenuated IPS and processed at the neural level during naturalistic interactions.

Higher-order social cognitive processes, namely mentalization and the adjustment of behaviors during social interactions, could also underlie attenuated behavioral IPS in autism. Autistic individuals have difficulties with mentalization^95,96^, which has been supported by behavioral and neuroimaging evidence^97–99^. Mentalization has been linked to behavioral IPS for non-autistic, but not autistic individuals, when they interacted with a virtual partner^45^. Yet, the extent to which mentalization lends to attenuated IPS in naturalistic interactions with autistic and non-autistic individuals is unknown. Difficulties making inferences about others’ behaviors, such as facial expressions or body language, could influence how one integrates subtle social cues and adapts their own behaviors in an interaction. Indeed, some findings demonstrate difficulties with inferences based on kinematic information^100^ and subtle facial expressions^101^ for autistic individuals. It is possible that autistic participants struggled to make inferences about the confederates’ behaviors and adapt their own behavior accordingly. Importantly though, social interactions are reciprocal, so difficulties inferring social cues and subsequent behavioral adaptation from one partner likely impact how the interaction partner predicts, infers, and adapts to behaviors as well. Thus, altered mentalizing abilities could lend to difficulties with mutual anticipation and adaptation of behaviors for both interaction partners and in turn influence the behavioral IPS that is produced.

Importantly, we aimed to assess whether differences in behavioral IPS between mixed dyads and non-autistic dyads might be linked to perceptual mechanisms. Thus, we did not evaluate this relationship in dyads including two autistic individuals. In a broader context of social interaction research in autism, some literature has focused on the relevance of dyad composition in relation to the Double Empathy hypothesis^102^. Some studies have found increased reported affiliative experiences for dyads with the same diagnosis compared to dyads with different diagnoses^103,104^. However, when employing objective measures, other studies have shown differences in behavioral IPS during collaborative interactions for dyads including an autistic individual, regardless of their interaction partner^17^, as well as evidence against an in-group advantage for autistic individuals when observing an autistic communication style^85^. This evidence is further supported by impression formation literature where less favorable impressions of autistic people have been reported by autistic and non-autistic observers and interaction partners^104,105^. Thus, our rigorous experimental approach could be extended to other such contexts in future studies to investigate the link between affiliative experience, behavioral IPS, and perception.

Finally, there are additional limitations to consider. Perceived IPS was subjectively reported and evaluated using a question following the interaction. This approach importantly allowed us to implicitly capture the extent to which participants perceived the temporal dynamics of the interaction with their partner. As previously mentioned, we cannot disentangle which modalities of IPS may have informed participants’ perception of such temporal dynamics. Moreover, it is also possible that autistic and non-autistic individuals may idiosyncratically consider different behavioral modalities to inform how they perceive IPS in social interactions. They may draw on different modalities or give more weight to certain modalities for informing their perception of the temporal dynamics of the interaction. We also cannot infer how participants conceptualized synchrony as they were not explicitly made aware about synchrony. It is possible that participants considered synchrony to mean similar engagement (i.e., our actions or ideas were similar) as opposed to simultaneous engagement (i.e., our actions were occurring at the same time). Future studies should consider increasing specificity for evaluating perceived IPS by including additional questions that assess which modalities of IPS may inform how one perceives the temporal dynamics of social interactions, as well as how synchrony was conceptualized. Furthermore, studies should also consider evaluating other synchrony modalities (e.g., facial expressions, neural, physiological), potentially leveraging machine-learning algorithms (e.g., OpenFace, OpenPose), to assess potential links with perceiving the temporal dynamics of the interaction in other modalities.

## Conclusion

The present study offers insight into the perceptual mechanisms of behavioral IPS in naturalistic social interactions of autistic and non-autistic individuals and speculates about additional cognitive mechanisms that may underlie attenuated behavioral IPS in autism. Our findings clarify that the perceived timing of others’ behaviors may not account for the behavioral attenuation of IPS in autism. While behavioral IPS expectedly differed between dyads with and without an autistic individual, perception of the temporal dynamics of the interaction partner’s behaviors and perception of event timing were not predictive of the behavioral IPS attenuation. Instead, other cognitive mechanisms, such as social attention, multisensory integration of verbal and non-verbal behaviors, as well as mentalization and mutual adaptation, may account for attenuated behavioral IPS in autism.

## Electronic supplementary material

Below is the link to the electronic supplementary material.

Supplementary Material 1

## Data Availability

A preregistration, scripts, and preprocessed data are available on OSF (https://osf.io/cw7n4). The perceptual simultaneity task script is available at https://github.com/aftmnelbier11/perceptual-simultaneity-task. The full dataset underlying this article may be shared after anonymization upon reasonable request to the corresponding authors (A.M.B, C.M.F.-W.). Supplementary Information is uploaded with the manuscript.
