# Supplementary material for: Attenuated behavioral interpersonal synchrony in autistic adults is not explained by perception of timing
Source: Sci Rep. 2025 Jun 20;15:20157. doi: 10.1038/s41598-025-05395-1 (PMC12181382; doi:10.1038/s41598-025-05395-1)

Attenuated behavioral interpersonal synchrony in autistic adults is not explained by perception of timing

Bierlich, A.M., Scheel, N.T., Koehler, J.C., Bloch, C., Plank, I.S.[+] & Falter-Wagner, C.M.[+]

Supplementary Information

S1. Confederates

Table S1.1 A frequency table detailing the number of autistic and non-autistic participants that each confederate interacted with.

Confederate	# of autistic interaction partners	# of non-autistic interaction partners
**1**	3	4
**2**	2	3
**3**	4	3
**4**	5	3
**5**	3	2
**6**	3	3
**7**	3	3
**8**	3	3
**9**	4	3
**10**	3	2

S2. Translated Perceived Synchrony and Rapport Questions

Table S2.1 The following questions were translated from English into German for use in the present study that was collected from a German-speaking sample. The questionnaire was merged from Cacioppo et al. [1] and Koehler et al. [2]. Questions were translated from Cacioppo et al. [1] as follows below. This questionnaire was also used in a student monography [3]. The questions pertaining to rapport and synchrony were also used in an fMRI study [4]. Concerning the questions pertaining to rapport and synchrony, the bolded question was used as an index of the perceived IPS rating referenced in the manuscript. The un-bolded questions were used to compute a mean composite rapport rating reported in Table 1 of the main text. The remaining two questions pertain to quality checks of the technical setup.

**Questions pertaining to rapport and synchrony**	
**English original**	**German translation (presently used)**
'How much rapport did you feel with your partner?'	'Wie sympathisch war Ihnen Ihr Partner?'
'How much did you trust your partner?'	'Wie sehr haben Sie Ihrem Partner vertraut?'
'How much did you like your partner?'	'Wie sehr mochten Sie Ihren Partner?'
**'How synchronized was the communication between you and your partner?'**	**'Wie synchron war die Kommunikation zwischen Ihnen und Ihrem Partner?'**
'How much would you like to work with your partner?'	'Wie gerne würden Sie mit Ihrem Partner zusammenarbeiten?'
'How much would you like to confide in your partner?'	'Wie gerne würden Sie Ihrem Partner etwas anvertrauen?'
'How close do you feel to your partner?'	'Wie nah fühlten Sie sich Ihrem Partner?'
**Questions pertaining to the technical setup**	
**English translation**	**German original (presently used)**
'How much did the video recording influence your behavior?**'**	'Wie sehr hat Sie die Videoaufnahme in Ihrem Verhalten beeinflusst?'
'How much did the plexiglass influence you in the interaction?'	'Wie sehr hat die Plexiglasscheibe Sie in der Interaktion beeinflusst?'

1. Cacioppo, S. et al. You are in sync with me: neural correlates of interpersonal synchrony with a partner. *Neuroscience.* **277**, 842-858. https://doi.org/10.1016/j.neuroscience.2014.07.051 (2014).
2. Koehler, J.C., Dong, M.S., Bierlich, A.M. et al. Machine learning classification of autism spectrum disorder based on reciprocity in naturalistic social interactions. Transl Psychiatry. **14**, 76. https://doi.org/10.1038/s41398-024-02802-5 (2024).
3. Scheel, N. Produced and perceived interpersonal synchrony, social affiliation, and affect in dyadic interactions of individuals with and without Autism Spectrum Disorder. (LMU München, 2023).
4. Bierlich, A. M., Plank, I. S., Scheel, N. T., Keeser, D., & Falter-Wagner, C. M. Neural processing of social reciprocity in autism. NeuroImage: Clinical. **46**, 103793. https://doi.org/10.1016/j.nicl.2025.103793 (2025).

S3. Quality check: reported influence of the video recording and plexiglass

Following the conversation task, participants were asked the extent to which being recorded (“Wie sehr hat Sie die Videoaufnahme in Ihrem Verhalten beeinflusst?”) and the presence of the plexiglass (“Wie sehr hat die Plexiglasscheibe Sie in der Interaktion beeinflusst?”) influenced their behaviors on a scale from 0 (‘gar nicht’ = ‘not at all’) to 3 (‘sehr’ = ‘very’).

Fig S3.1 The bar graph depicts the extent to which being recorded influenced their behaviors.

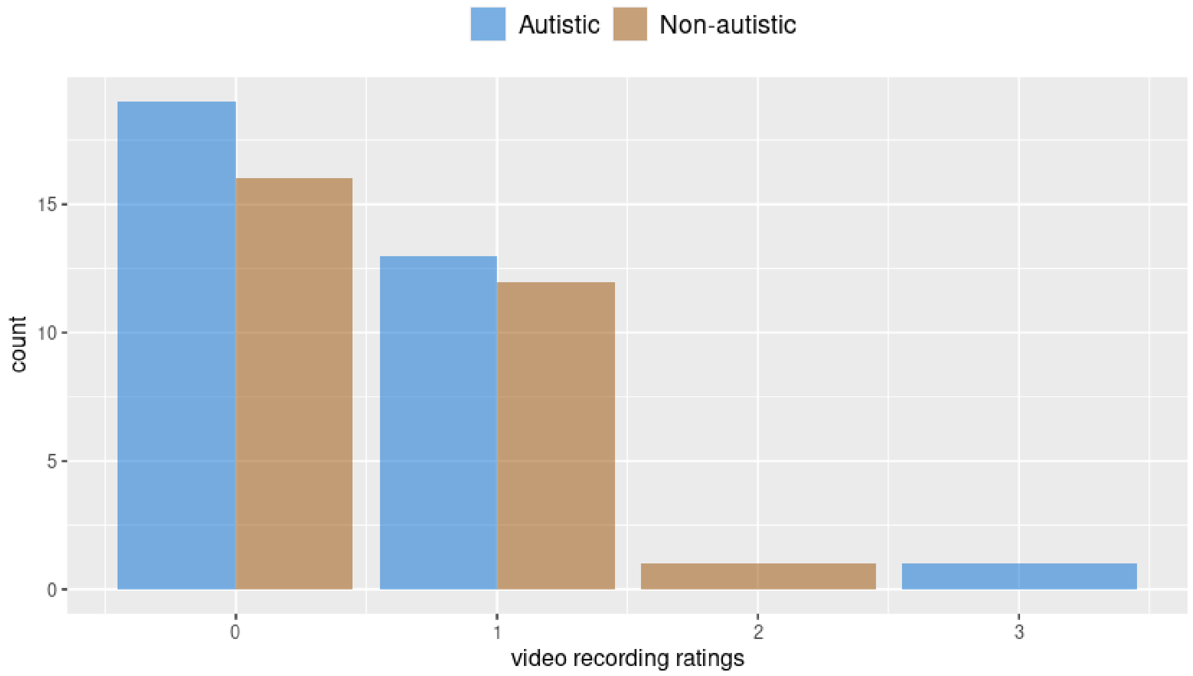

Fig S3.2 The bar graph depicts the extent to which the plexiglass influenced their behaviors.

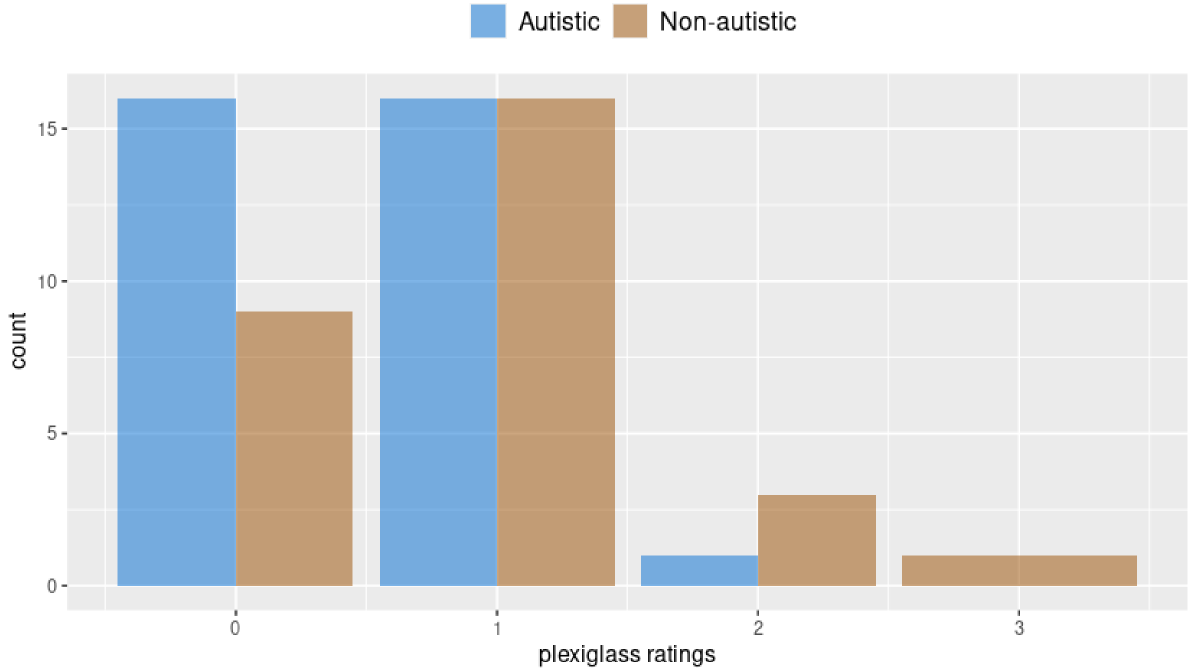

S4. Behavioral IPS, pseudo-synchrony, and motion quantity comparisons for all ROIs

S4.1 Behavioral IPS

Although head motion IPS was the focus of the present analysis, behavioral IPS for the body and total ROIs are also visualized in Fig S4.1.

Fig S4.1 The box- and jitter plot depict raw produced IPS values for mixed dyads including an autistic participant (blue) and non-autistic dyads (brown) for each ROI (body, head, total) within each task (h: hobbies; m: meal-planning).

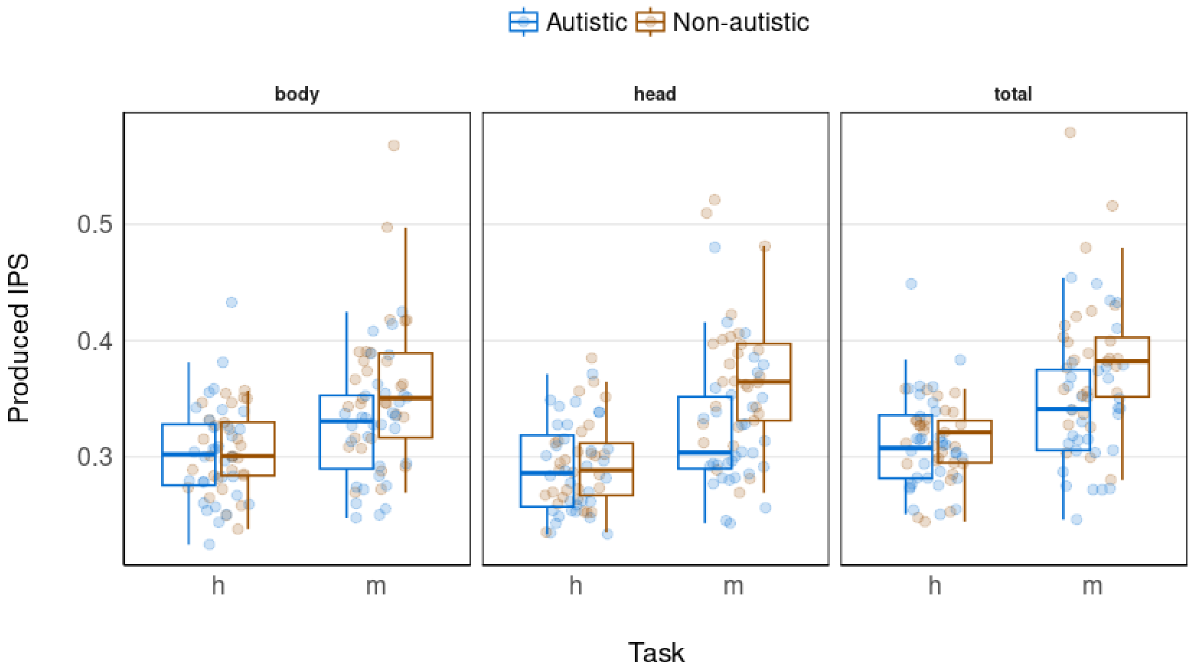

S4.2 Pseudo-synchrony

Moreover, pseudo-synchrony values were computed and comparatively evaluated to ensure that the behavioral IPS values were above chance level. This was accomplished using the shuffle() function in rMEA. Motion energy timeseries were randomly shuffled to create 1000 pseudo-dyads for each task, while maintaining the relative position of the timeseries to avoid pairing two confederates. The same procedure reported in the manuscript for computing true behavioral IPS cross-correlation values was used. The differences between true and pseudo-synchrony are visualized in Fig S4.2.1 – Fig S4.2.3. A Bayesian one-sample t-test was conducted considering the theoretical mean of pseudo-synchrony (μ) against the behavioral IPS to evaluate pseudo-synchrony in the head ROI. There was decisive evidence in favor of differences between head movement IPS (hobbies: BF10 = 1366.987; meal-planning: BF10 = 2.530 x 108).

Fig S4.2.1 The density histogram depicts the distribution of the raw produced IPS values (light blue), with the mean produced IPS (blue) and the mean pseudo-synchrony value (red) for the **head** ROI in the hobbies (a) and meal-planning (b) tasks.

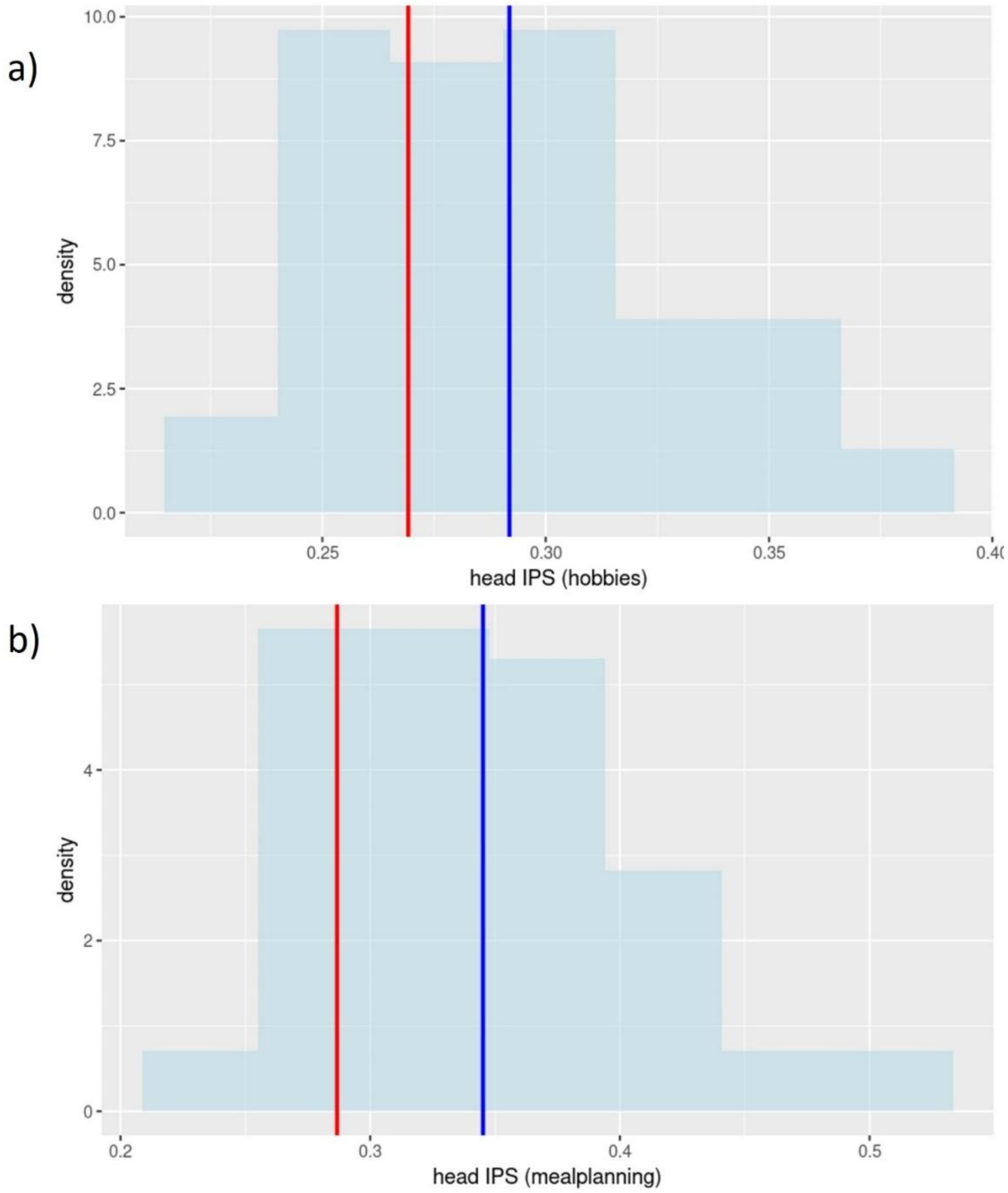

Fig S4.2.2 The density histogram depicts the distribution of the raw produced IPS values (light blue), with the mean produced IPS (blue) and the mean pseudo-synchrony value (red) for the **body** ROI in the hobbies (a) and meal-planning (b) tasks.

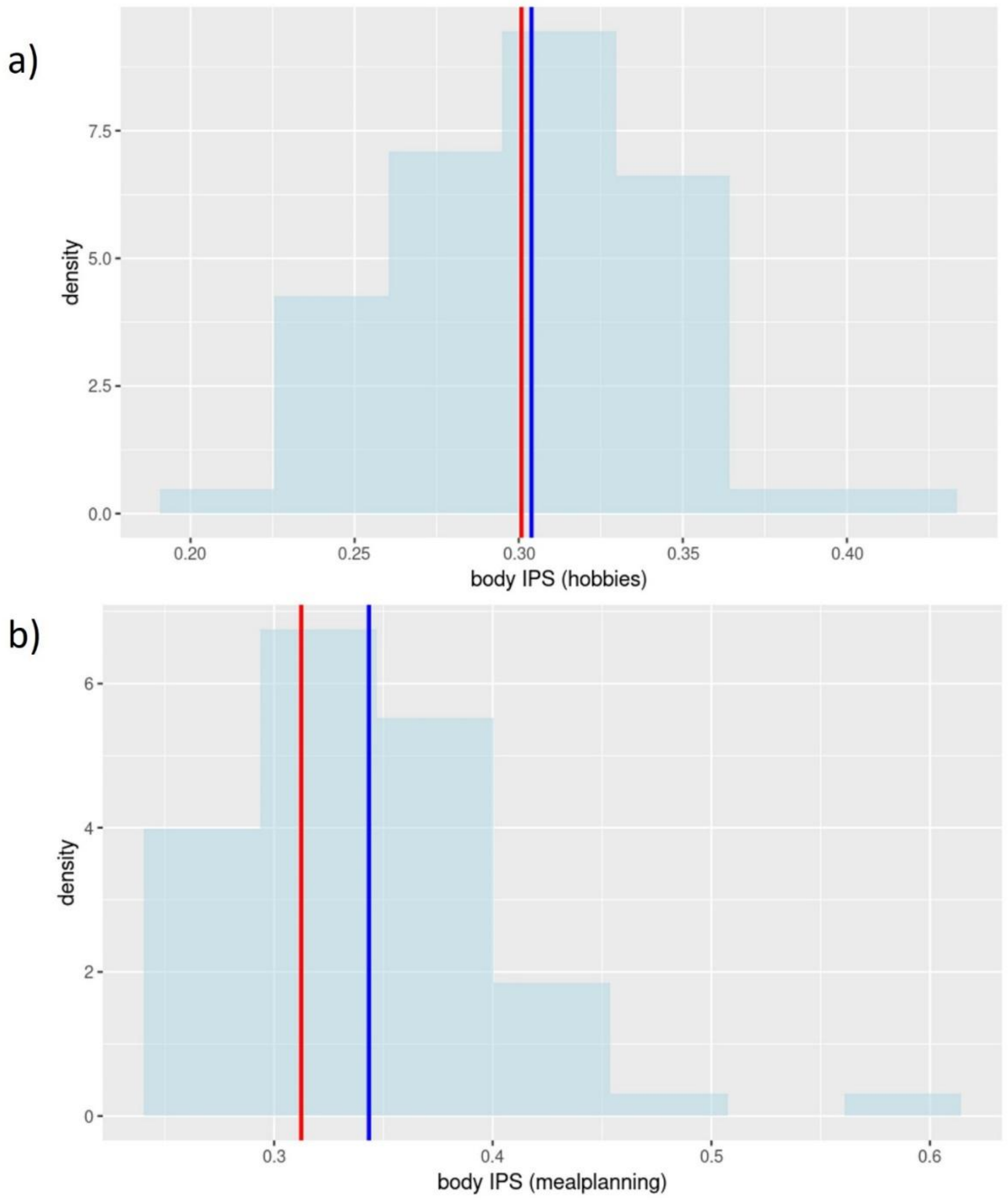

Fig S4.2.3 The density histogram depicts the distribution of the raw produced IPS values (light blue), with the mean produced IPS (blue) and the mean pseudo-synchrony value (red) for the **total** ROI in the hobbies (a) and meal-planning (b) tasks.

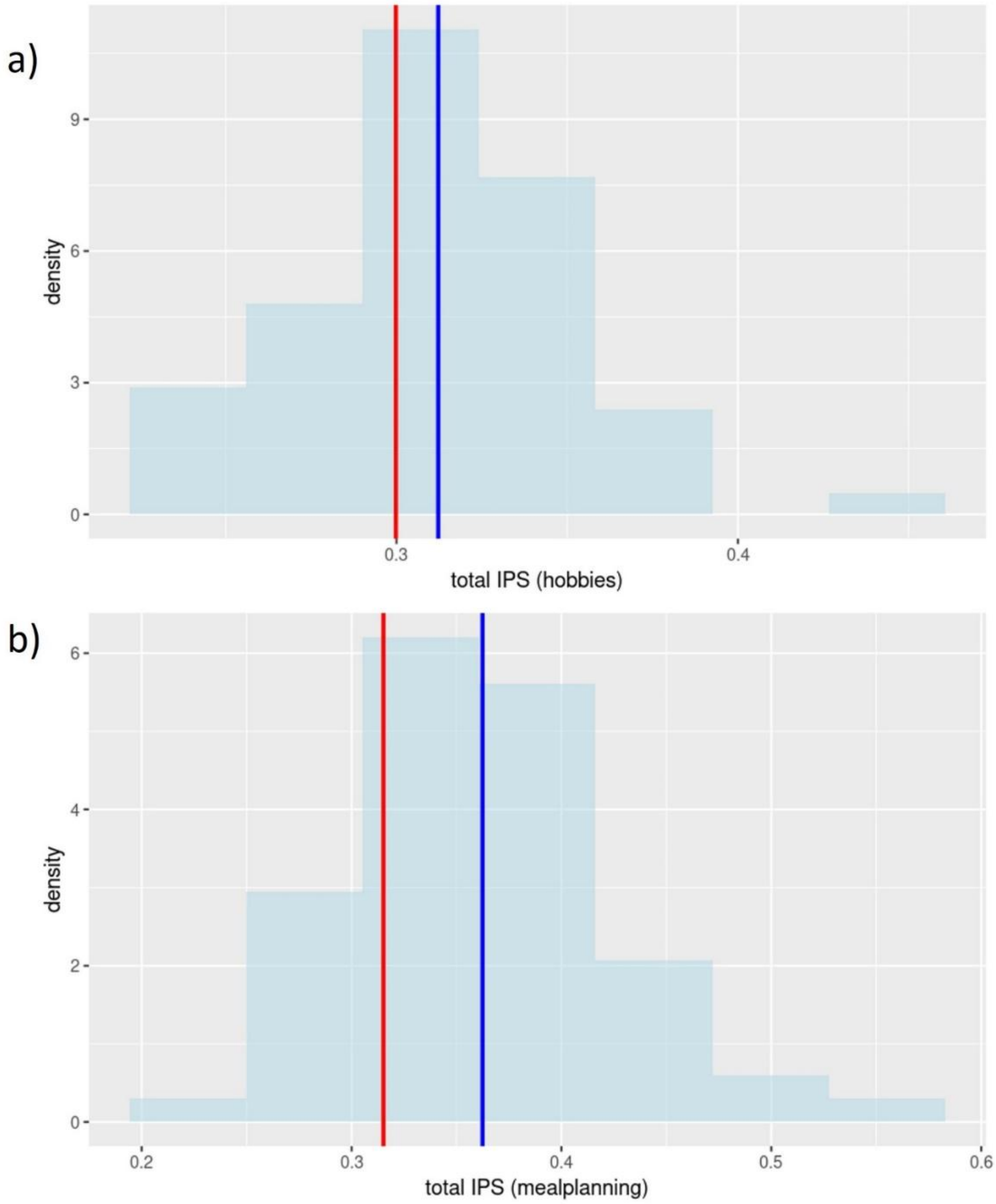

S4.3 Dyadic motion quantity

Dyadic motion quantity was also calculated from the absolute values of movement quantity within a dyad. Dyadic motion quantity for the head ROI was included as a covariate in the analysis. Although head motion was the focus of the present analysis, motion quantity for the body and total ROIs are visualized in Fig S4.3.

Fig S4.3 The box- and jitter plot depict raw motion quantity values for mixed dyads including an autistic participant (blue) and non-autistic dyads (brown) participants for each ROI (body, head, total) within each task (h: hobbies; m: meal-planning).

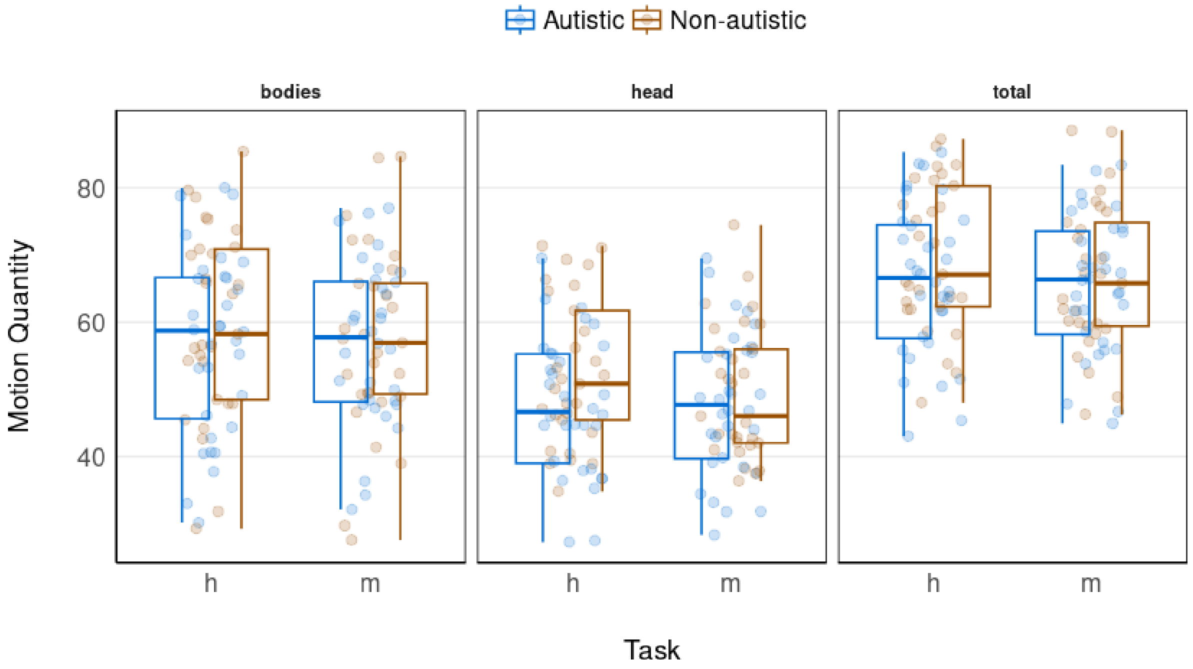

S5. SBC prior and posterior predictive checks from the first model

Fig S5.1 The prior predictive distribution from the simulated datasets for the first model reported in the manuscript.

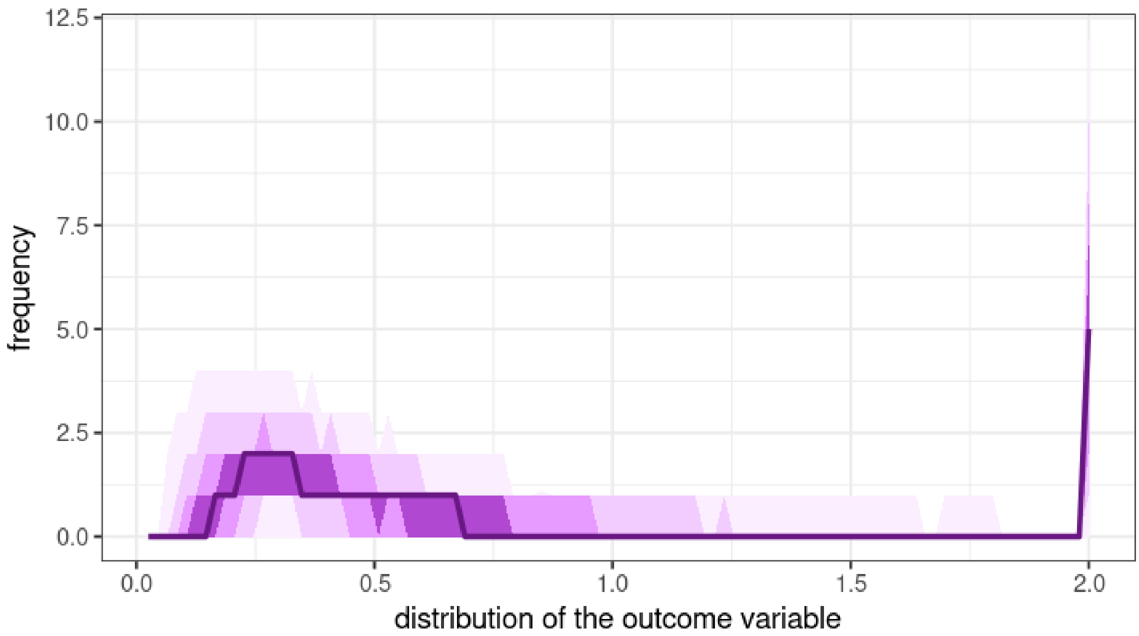

Fig S5.2 The posterior predictive checks from all simulated datasets for the first model reported in the manuscript.

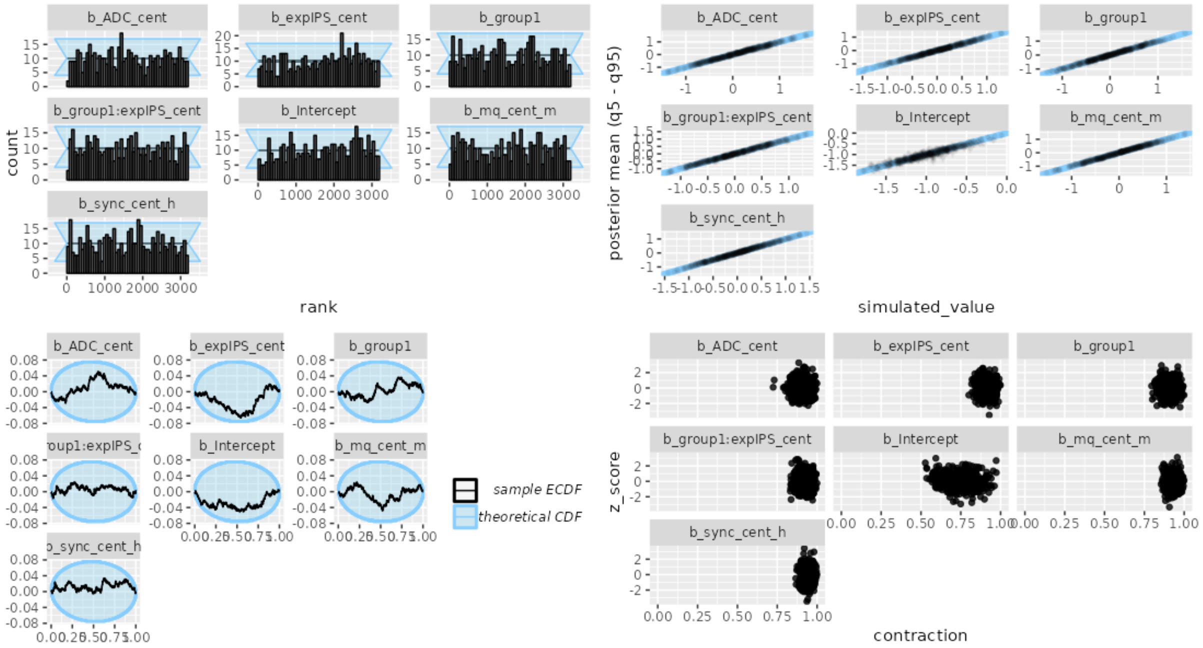

S6. Posterior predictive checks from the first model

Fig S6.1 The posterior predictive checks of the first model reported in the manuscript. The distribution of the true data (y) overlaid on the predicted data (y_{rep}). The model is not a perfect fit but deemed suitable for the present analysis considering the distribution of the true data.

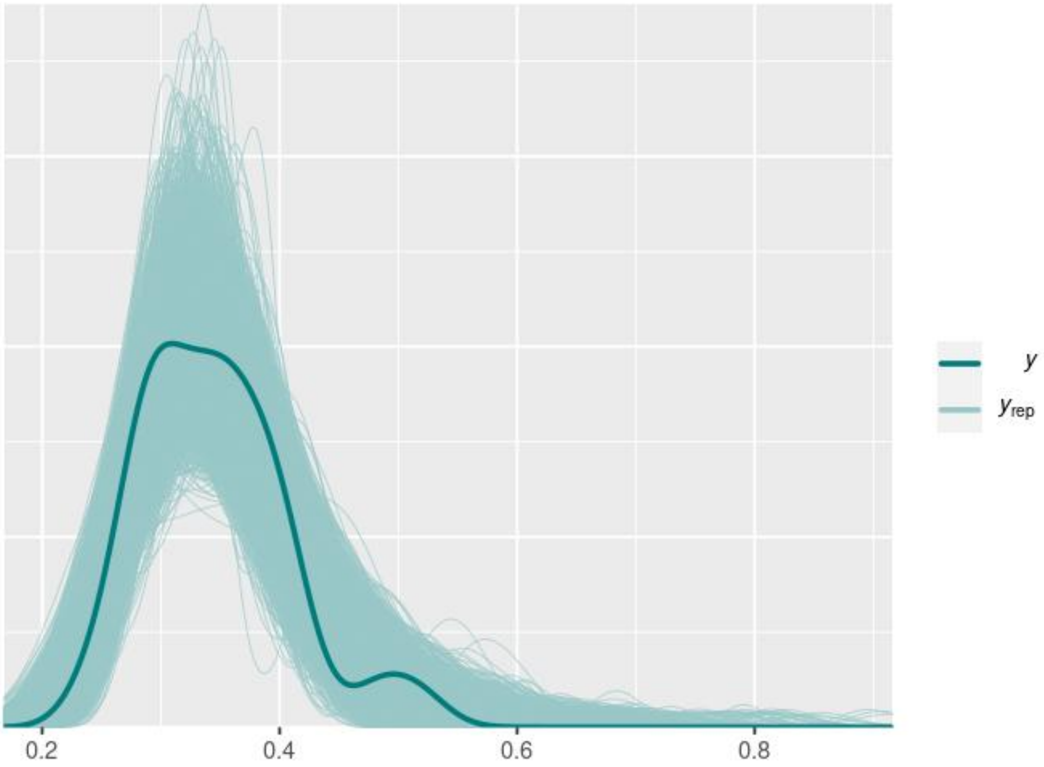

S7. Reverse model with behavioral IPS from the hobbies task

For completion purposes, a reverse model with behavioral IPS from the hobbies task as the outcome and behavioral IPS from the meal-planning task as a predictor. The same procedure, priors, model parameters, and remaining predictors were used as with the first model reported in the manuscript. The posterior predictive checks are depicted in Fig S7.1, and the posterior estimates are shown in Fig S7.2. The only credible association was between behavioral IPS from the hobbies and meal-planning tasks. There was no credible difference between groups, demonstrating the hobbies task as a suitable baseline task.

Fig S7.1 The posterior predictive checks of the model run on the true data. The distribution of the true data (y) overlaid on the predicted data (y_{rep}).

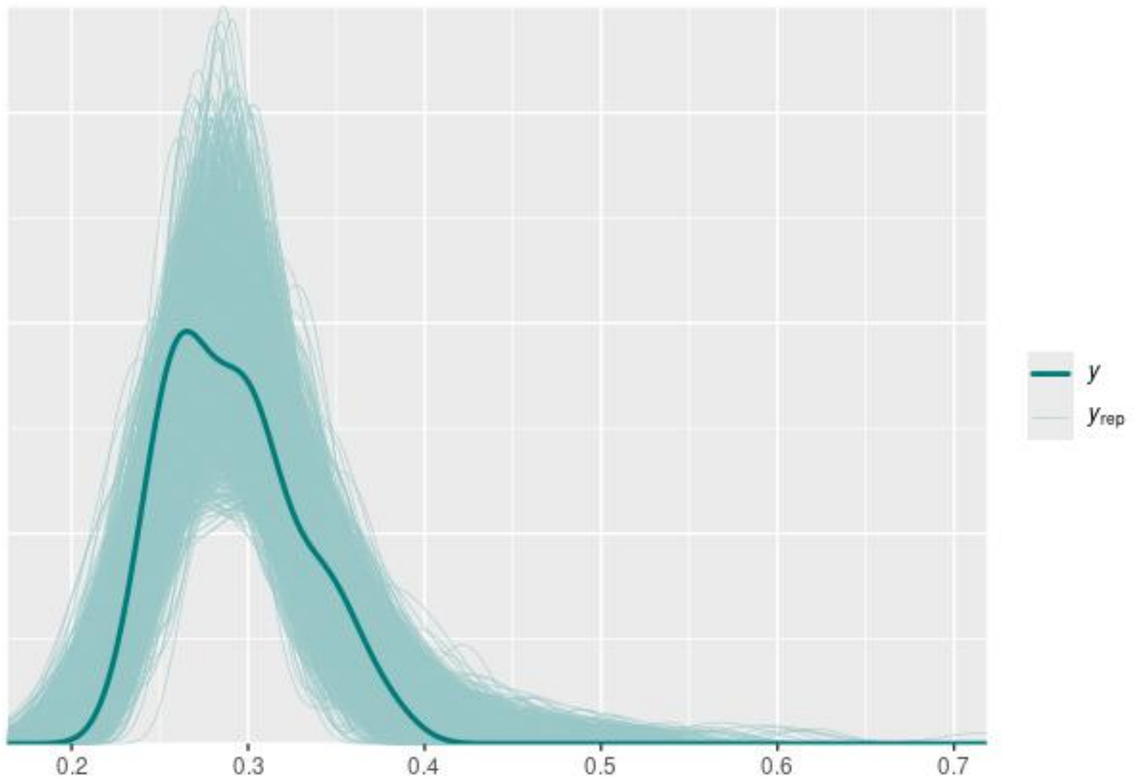

Fig S7.2 Posterior predictive distributions of the estimates for each predictor: group, perceived IPS, their interaction, motion quantity, self-reported dyspraxia symptoms, and dyadic behavioral IPS from the cooperative meal-planning task (behavioral IPSm). The thin, bold lines reflect the 95% and 66% intervals of the distributions. The dotted line reflects the zero grand average estimate. Credible effects are visualized as those that do not cross the zero grand average estimate. With this model, only behavioral IPS from the meal-planning task was credibly associated with behavioral IPS from the hobbies task.

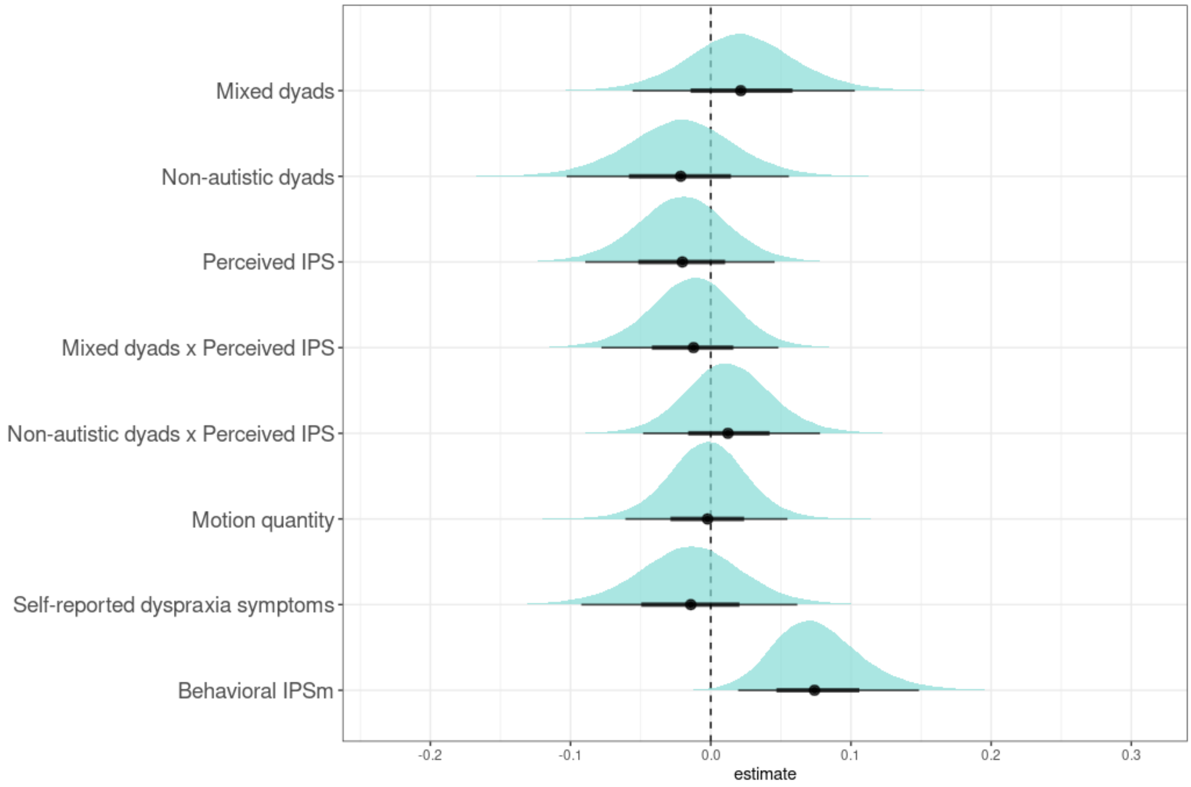

S8. Preregistered models

Supplementary Material S8 reports the Bayesian linear mixed models that were initially preregistered for the sake of transparency.

```
# Behavioral IPS
priors.bIPS
```

```
##               prior     class coef group resp dpar nlpar   lb   ub source
##  normal(-1.2, 0.2) Intercept                             <NA> <NA>   user
##   normal(0.2, 0.1)     sigma                             <NA> <NA>   user
##    normal(0, 0.25)         b                             <NA> <NA>   user
##    normal(0, 0.25)        sd                             <NA> <NA>   user
##     normal(0, 0.1)       ndt                             <NA> <NA>   user
```

```
summary(mod.bIPS)
```

```
##  Family: shifted_lognormal
##   Links: mu = identity; sigma = identity; ndt = identity
## Formula: sync_head ~ group * task + mq_cent + (1 | Confederate)
##    Data: df.long (Number of observations: 122)
##   Draws: 4 chains, each with iter = 40000; warmup = 8000; thin = 1;
##          total post-warmup draws = 128000
##
## Multilevel Hyperparameters:
## ~Confederate (Number of levels: 10)
##               Estimate Est.Error l-95% CI u-95% CI Rhat Bulk_ESS Tail_ESS
## sd(Intercept)     0.08      0.04     0.02     0.17 1.00    33131    31442
##
## Regression Coefficients:
##              Estimate Est.Error l-95% CI u-95% CI Rhat Bulk_ESS Tail_ESS
## Intercept       -1.52      0.16    -1.84    -1.22 1.00    37153    42840
## group1          -0.05      0.02    -0.09    -0.01 1.00    98388    79586
## task1           -0.12      0.03    -0.17    -0.07 1.00    54146    70643
## mq_cent          0.03      0.02    -0.01     0.07 1.00   102127    80305
## group1:task1     0.05      0.02     0.01     0.09 1.00   105057    80904
##
## Further Distributional Parameters:
##       Estimate Est.Error l-95% CI u-95% CI Rhat Bulk_ESS Tail_ESS
## sigma     0.20      0.03     0.14     0.27 1.00    37869    52121
## ndt       0.09      0.03     0.02     0.15 1.00    35567    41748
##
## Draws were sampled using sample(hmc). For each parameter, Bulk_ESS
## and Tail_ESS are effective sample size measures, and Rhat is the potential
## scale reduction factor on split chains (at convergence, Rhat = 1).
```

```
# perceived IPS
priors.pIPS
```

```
##             prior     class coef group resp dpar nlpar   lb   ub source
##  normal(-1.07, 1) Intercept    1                       <NA> <NA>   user
##  normal(-0.57, 1) Intercept    2                       <NA> <NA>   user
##  normal(-0.18, 1) Intercept    3                       <NA> <NA>   user
##   normal(0.18, 1) Intercept    4                       <NA> <NA>   user
##   normal(0.57, 1) Intercept    5                       <NA> <NA>   user
##   normal(1.07, 1) Intercept    6                       <NA> <NA>   user
##    normal(0, 0.5)         b                            <NA> <NA>   user
##    normal(0, 0.5)        sd                            <NA> <NA>   user
##            lkj(2)       cor                            <NA> <NA>   user
```

```
summary(mod.pIPS)
```

```
##  Family: cumulative
##   Links: mu = probit; disc = identity
## Formula: PS_score ~ group + sync_log_m + sync_log_h + group:sync_log_m + group:sync_log_h + (group | Confederate)
##    Data: df (Number of observations: 61)
##   Draws: 4 chains, each with iter = 40000; warmup = 8000; thin = 1;
##          total post-warmup draws = 128000
##
## Multilevel Hyperparameters:
## ~Confederate (Number of levels: 10)
##                         Estimate Est.Error l-95% CI u-95% CI Rhat Bulk_ESS
## sd(Intercept)               0.37      0.21     0.02     0.83 1.00    41569
## sd(group1)                  0.32      0.20     0.02     0.77 1.00    44071
## cor(Intercept,group1)       0.14      0.42    -0.71     0.85 1.00    91068
##                         Tail_ESS
## sd(Intercept)              51690
## sd(group1)                 54089
## cor(Intercept,group1)      90664
##
## Regression Coefficients:
##                     Estimate Est.Error l-95% CI u-95% CI Rhat Bulk_ESS Tail_ESS
## Intercept[1]           -2.19      0.36    -2.94    -1.53 1.00    94014    82453
## Intercept[2]           -1.48      0.27    -2.04    -0.97 1.00   123850   101992
## Intercept[3]           -0.98      0.24    -1.45    -0.52 1.00   134151   109593
## Intercept[4]           -0.49      0.22    -0.91    -0.04 1.00   127711   108621
## Intercept[5]            0.32      0.22    -0.09     0.79 1.00   103244   101243
## Intercept[6]            1.04      0.24     0.60     1.56 1.00    95461   102748
## group1                 -0.37      0.18    -0.73    -0.01 1.00   100889    88014
## sync_log_m              0.03      0.17    -0.30     0.35 1.00   112904    94031
## sync_log_h             -0.11      0.15    -0.40     0.18 1.00   137777   100903
## group1:sync_log_m      -0.03      0.17    -0.36     0.30 1.00   120302    96803
## group1:sync_log_h      -0.06      0.15    -0.35     0.23 1.00   136074   100174
##
## Further Distributional Parameters:
##      Estimate Est.Error l-95% CI u-95% CI Rhat Bulk_ESS Tail_ESS
## disc     1.00      0.00     1.00     1.00   NA       NA       NA
##
## Draws were sampled using sample(hmc). For each parameter, Bulk_ESS
## and Tail_ESS are effective sample size measures, and Rhat is the potential
## scale reduction factor on split chains (at convergence, Rhat = 1).
```

S9. Group comparisons of all predictors

Fig S9.1 The rain plots depict the raw values for each predictor for each group. Where individual measures are shown, the group comparison is between autistic and non-autistic participants. Where dyadic measures are shown, the group comparison is between mixed and non-autistic dyads. A table of the means and test statistics is reported in the manuscript.

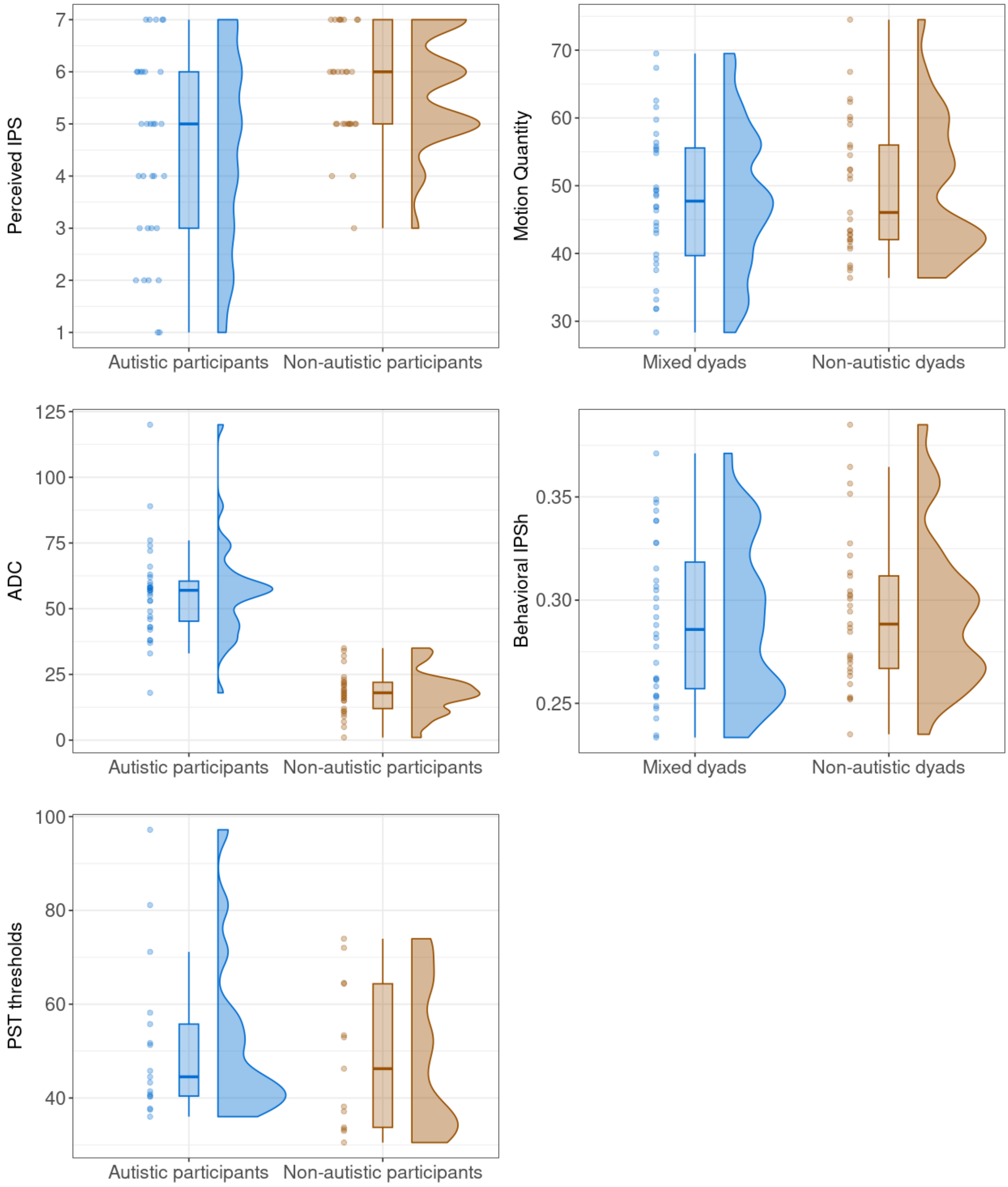

S10. Model comparison using leave-one-out cross validation

Fig S10.1 The model comparisons from the leave-one-out cross validation are reported, including the elpd difference and elpd standard error for each comparison. All models include confederate as a random intercept. The best model is shown first with all other models in comparison. A model including group and behavioral IPS from the hobbies task (IPSh) best explains the data. This model is credibly better than models including perceived IPS ratings (pIPS), the intercept model, motion quantity (mq), self-reported dyspraxia symptoms (ADC), and the combination of the three predictors. An elpd difference greater than 4 and elpd standard error greater than 2 is considered credible. Models that are credibly less favorable from the best model are bolded and outlined in grey.

model	elpd_diff	se_diff
group + IPSh	0	0
group + mq + IPSh	- 0.07887	1.246832
group + pIPS + mq + IPSh	- 0.9477	1.14144
group + mq + ADC + IPSh	- 1.07168	1.432487
group + ADC + IPSh	- 1.07431	0.481759
group + pIPS + IPSh	- 1.14047	0.428701
group + pIPS + mq + ADC + IPSh	- 1.83507	1.338199
group + pIPS + ADC + IPSh	- 2.13638	0.671614
group + mq	- 3.10343	3.049805
group	- 3.20388	3.025804
group + mq + ADC	- 3.82427	3.022071
group + pIPS + mq	- 3.96399	3.005175
group + ADC	- 4.06227	2.971753
group + pIPS	- 4.17659	3.026543
ADC + IPSh	- 4.50533	2.974304
mq + ADC + IPSh	- 4.68462	3.201394
group + pIPS + mq + ADC	- 4.7603	2.966636
group + pIPS + ADC	- 5.07733	2.992403
pIPS + mq + ADC + IPSh	- 5.24744	2.791018
pIPS + mq + IPSh	- 5.35455	2.841051
pIPS + ADC + IPSh	- 5.52982	2.784781
mq + IPSh	- 6.11652	3.742855
pIPS + IPSh	- 6.49379	3.210842
IPSh	- 6.67598	3.739122
mq + ADC	- 7.02105	3.752448
ADC	- 7.0989	3.742376
pIPS + mq + ADC	- 7.79351	3.532714
pIPS + ADC	- 8.09487	3.658323
pIPS + mq	- 8.66293	3.809517
mq	- 8.95381	4.475075
Intercept	- 9.73522	4.569048
pIPS	- 9.96411	4.166783

S11. SBC prior and posterior predictive checks from the second model

The predictive prior distribution and posterior predictive checks are depicted in Fig S11.1 and Fig S11.2. The posterior predictive checks are depicted in Fig S11.3, and the posterior estimates are shown in Fig S11.4.

Fig S11.1 The prior predictive distribution of the simulated datasets for the model including simultaneity thresholds.

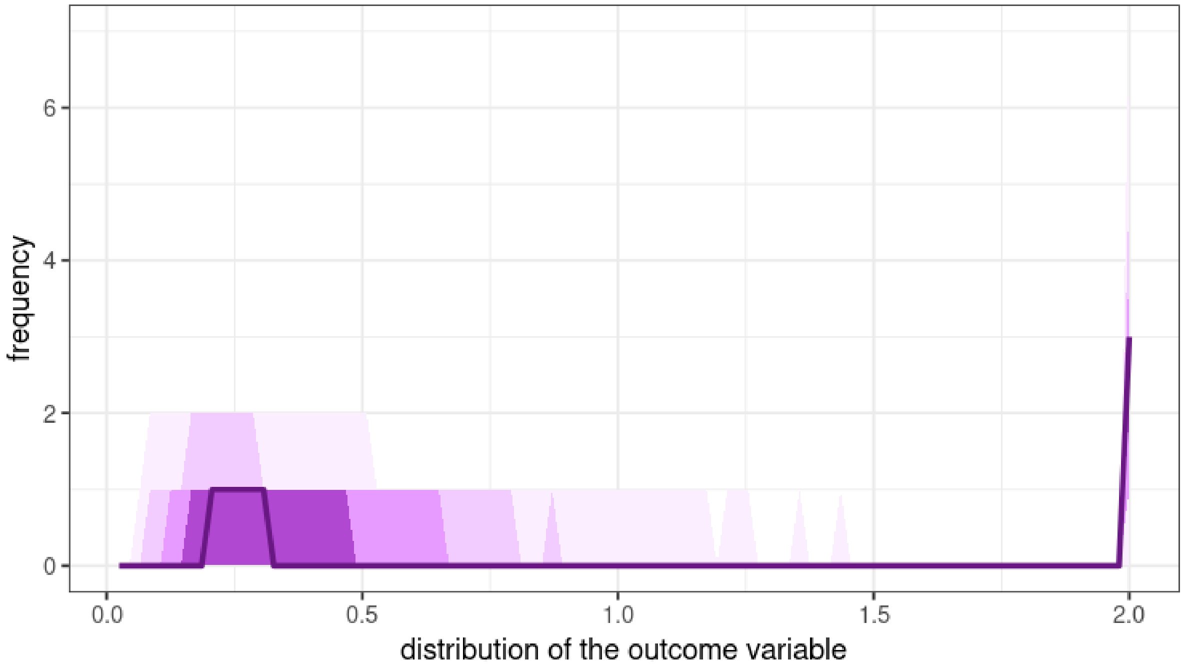

Fig S11.2 The posterior predictive checks from all simulated datasets for the model including simultaneity thresholds.

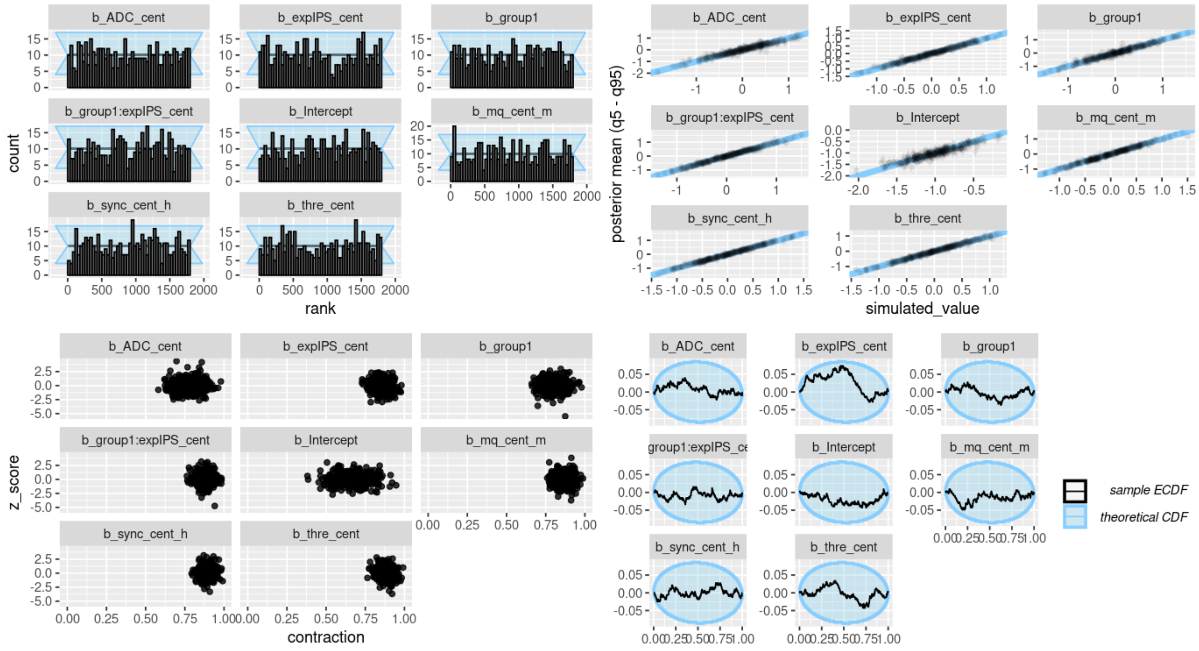

Fig S11.3 The posterior predictive checks of the model run on the true data. The distribution of the true data (y) overlaid on the predicted data (y_{rep}).

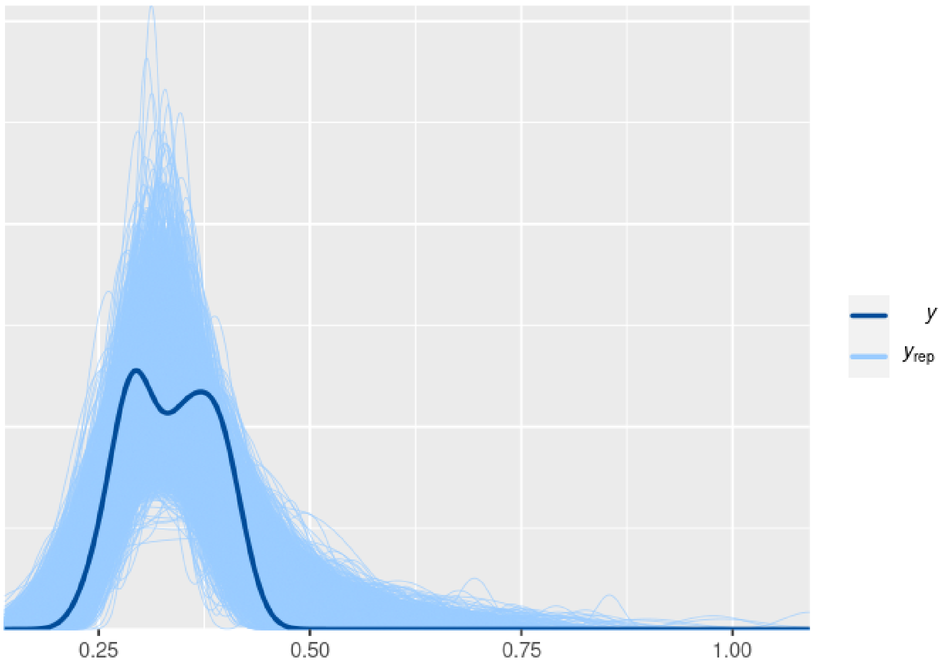

Fig S11.4 Posterior predictive distributions of the estimates for each predictor: group, perceived IPS, their interaction, event timing perception (PST), motion quantity, self-reported dyspraxia symptoms, and baseline behavioral IPS from the hobbies task (behavioral IPSh). The thin, bold lines reflect the 95% and 66% intervals of the distributions. The dotted line reflects the zero grand average estimate. Credible effects are visualized as those that do not cross the zero grand average estimate. With this model, no credible effects were found to predict behavioral IPS.

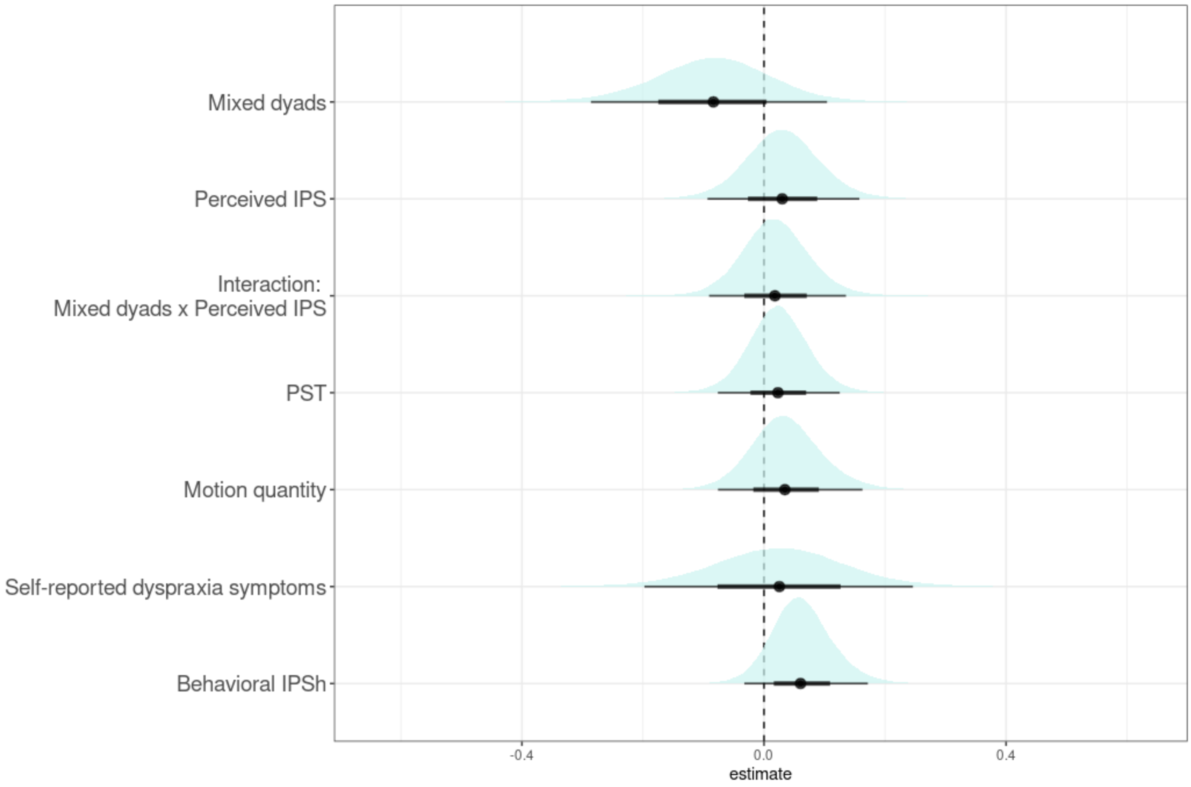

Supplement: Supplementary file 1 — Supplementary Material 1 [file 41598_2025_5395_MOESM1_ESM.pdf]
